# Malignant transformation of vaginal adenosis to clear cell carcinoma without prenatal diethylstilbestrol exposure: a case report and literature review

**DOI:** 10.1186/s12885-019-6026-1

**Published:** 2019-08-13

**Authors:** Lihong Pang, Lei Li, Lan Zhu, Jinghe Lang, Yalan Bi

**Affiliations:** 1 Department of Obstetrics and Gynecology, Peking Union Medical College Hospital, Peking Union Medical College & Chinese Academy of Medical Science, Shuaifuyuan No. 1, Dongcheng District, Beijing, 100730 China; 2 Department of Pathology, Peking Union Medical College Hospital, Peking Union Medical College & Chinese Academy of Medical Science, Beijing, 100730 China

**Keywords:** Vaginal adenosis, Vaginal clear cell carcinoma, Pathology, Cytology, Radiotherapy

## Abstract

**Background:**

We report an extremely rare case of vaginal clear cell carcinoma, which originated from the malignant transformation of vaginal adenosis without prenatal diethylstilbestrol (DES) exposure.

**Case presentation:**

In this case, the patient was a Chinese woman with a history of two decades of intermittent vaginal pain, sexual intercourse pain and vaginal contact bleeding. On September 1, 2011, when the patient was 39 years old, a vaginal biopsy revealed vaginal adenosis. After intermittent drug and laser treatment, her symptoms did not improve. Four years later, on March 4, 2015, another vaginal biopsy for abnormal vaginal cytology revealed atypical vaginal adenosis. After treatment with sirolimus, her symptoms and abnormal vaginal cytology results persisted, and she underwent laparoscopic hysterectomy with bilateral salpingo-oophorectomy and excision of the vaginal lesions. One year after the hysterectomy, on August 15, 2017, the vaginal cytology results suggested atypical glandular cells, and a biopsy revealed vaginal clear cell carcinoma originating from the atypical vaginal adenosis. A wide local resection of the vaginal lesions was performed, followed by concurrent chemoradiotherapy. Regular follow-up over 16 months showed no evidence of the recurrence of vaginal adenosis or cancer.

**Conclusions:**

Based on the evolution of a series of pathological evidence, we report the fourth case in the world of vaginal clear cell carcinoma originating from vaginal adenosis without prenatal DES exposure. Wide local excision with radiotherapy provided at least 16 months of disease-free survival.

## Background

Vaginal adenosis is defined as the presence of residual Mullerian ducts, which are considered remnants of the accessory mesonephric duct from the embryonic period [1], in the vaginal wall and superficial stroma of the vagina after complete vaginal development [2]. The persistence of Mullerian cells altered at the subcellular level could form the basis for the development of carcinoma in later life with a history of maternal ingestion of estrogens [3]. In November 1971, an association of the use of diethylstilbestrol (DES) during pregnancy with the subsequent development of vaginal adenocarcinoma in exposed offspring was announced [4]. Numerous studies and databases have reported and registered cases of vaginal and cervical clear cell carcinoma originating from vaginal adenosis caused by DES. However, primary vaginal clear cell carcinoma without prenatal DES exposure is very rare. To the best of our knowledge, there have only been three cases of vaginal clear cell carcinoma due to the potential malignant transformation of vaginal adenosis or atypical vaginal adenosis without prenatal DES exposure in the English literature [5–7]. In this study, we report the fourth case and review the relevant studies in the literature.

## Case presentation

The patient in this report provided consent for its publication. The Institutional Review Board of Peking Union Medical College Hospital approved this study. The patient was a 45-year-old postmenopausal Han Chinese woman, gravida 5, para 2, who presented with intermittent vaginal pain, sexual intercourse pain and contact vaginal bleeding for 20 years. Her menstruation was regular with mild dysmenorrhea and a visual analog scale score of 4 of 10. Details of the diagnosis and treatments are listed in Table 1. She had absolutely no prenatal exposure to DES or any other type of estrogen. DES was never introduced into the Chinese market, and her parents stated that they did not have access to it during the Cold War, which was an era of prevalent DES use.

**Table 1:** Chronicle of the diagnosis and treatment. HPV, human papillomavirus

Date	Procedures of diagnosis and treatment	Pathological findings
September 1, 2011	Vaginal biopsy	Vaginal adenosis
December 16, 2011	Vulvar biopsy	Chronic inflammation; absence of focal epithelial absence; granulation tissue formation
March 4, 2013	Cytology	A few atypical glandular cells and high-grade squamous intraepithelial lesion
March 4, 2013	High-risk HPV test	Negative
April 10, 2013	Vaginal and cervical biopsy	chronic inflammation; cervical intraepithelial neoplasia of grade I
May 10, 2013	Fractional curettage	Endometrium of late proliferative phase
March 4, 2015	Cytology	Atypical squamous cells: cannot exclude high-grade squamous intraepithelial lesion
March 4, 2015	Vaginal biopsy	Vaginal adenosis; moderate atypical hyperplasia of focal squamous epithelium
December 24, 2015	Cytology	A few atypical gland cells
December 24, 2015	High-risk HPV test	Negative
March 18, 2016	Cytology	Suspicious adenocarcinoma of cervix; atypical squamous epithelial cells of vagina
March 3, 2016	Fractional curettage	A little cervical canal tissue and endometrium of secretory phase
April 15, 2016	Vaginal biopsy	The serous papillary glands with active growth; chronic inflammation
May 4, 2016	Hysterectomy with bilateral salpingoophorectomy, and excision of vaginal lesions	Normal findings except atypical vaginal adenosis in the vaginal wall
May 15, 2017	Cytology	A few atypical gland cells
May 15, 2017	Biopsy of vaginal stump	Serous papillary glands with active growth, which suggested atypical adenosis
August 15, 2017	Excision of vaginal lesions	The mass of mid-anterior vaginal wall was confirmed to be clear cell carcinoma
September 15, 2017	Wide local resection of vaginal lesions	Atypical vaginal adenosis with negative incision margin
July 18, 2018	Biopsy of vulvar ulcer	Chronic inflammation of fibrous tissue and squamous epithelium

### Discovery and treatment of vaginal adenosis (September 2011 to December 2015)

**Fig. 1:**
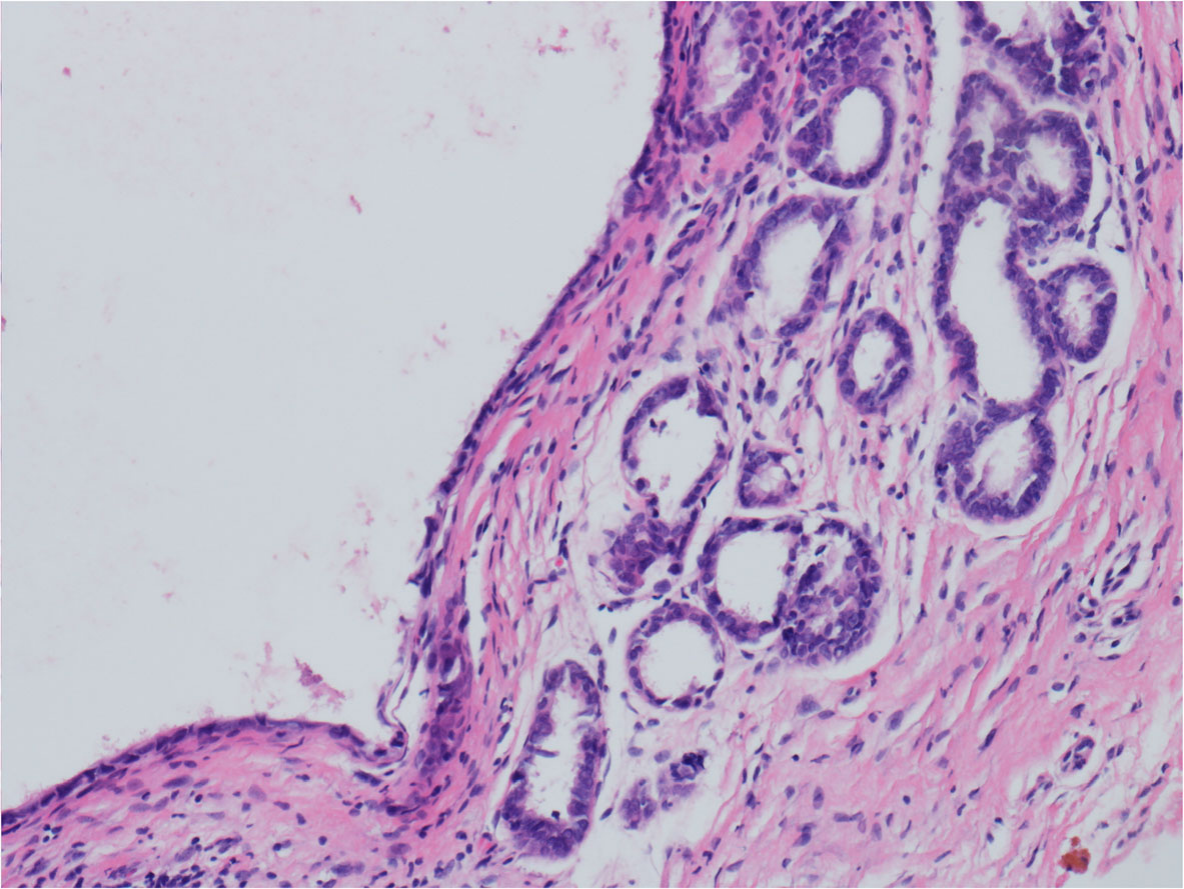
Vaginal biopsy on March 4, 2015 revealed vaginal adenosis (hematoxylin and eosin staining, × 10)

On September 1, 2011, at age 39, the patient underwent a vaginal biopsy due to a vaginal ulcer found through physical examination. The pathological findings revealed vaginal adenosis. After 3 months of treatment with tacrolimus, the ulcerative lesion persisted. A biopsy of a 2-cm hypopigmented area of the medial right minor labia was performed, and the pathological findings revealed chronic inflammation with granulation formation. Later, two laser treatments were performed for the vaginal adenosis and vulvar lesions, and remission was achieved after the treatment. On March 4, 2013, the patient went to the outpatient clinic due to aggravated vaginal pain. On physical examination, her bilateral minor labia were slightly edematous with thinned mucosa, but the vagina appeared normal. A cervical cytology test revealed a high-grade squamous intraepithelial lesion (HSIL), and her high-risk human papillomavirus (HPV) test result was negative. Subsequently, a cervical biopsy and fractional curettage revealed grade I cervical intraepithelial neoplasia and normal endometrium of the late proliferative phase. No further surgical interventions were performed, such as loop electrosurgical excision or conization. She underwent 2 months of treatment with sirolimus (rapamycin). On March 4, 2015, she came to the hospital due to vaginal pain and an inability to have sexual intercourse. A physical examination revealed that the lower third of the vaginal mucosa was swollen with an erosive lesion 0.5 cm in diameter. Her cervical cytology results showed ASC-H (atypical squamous cells, cannot exclude HSILs). A biopsy revealed vaginal adenosis with moderate atypical hyperplasia of the focal squamous epithelium (Fig. 1). She was treated with sirolimus for another two months. The symptoms did not improve; she stopped taking the medicine and was transferred to the unit of the authors.

### Discovery and treatment of atypical vaginal adenosis (December 2015 to August 2017)

**Fig. 2:**
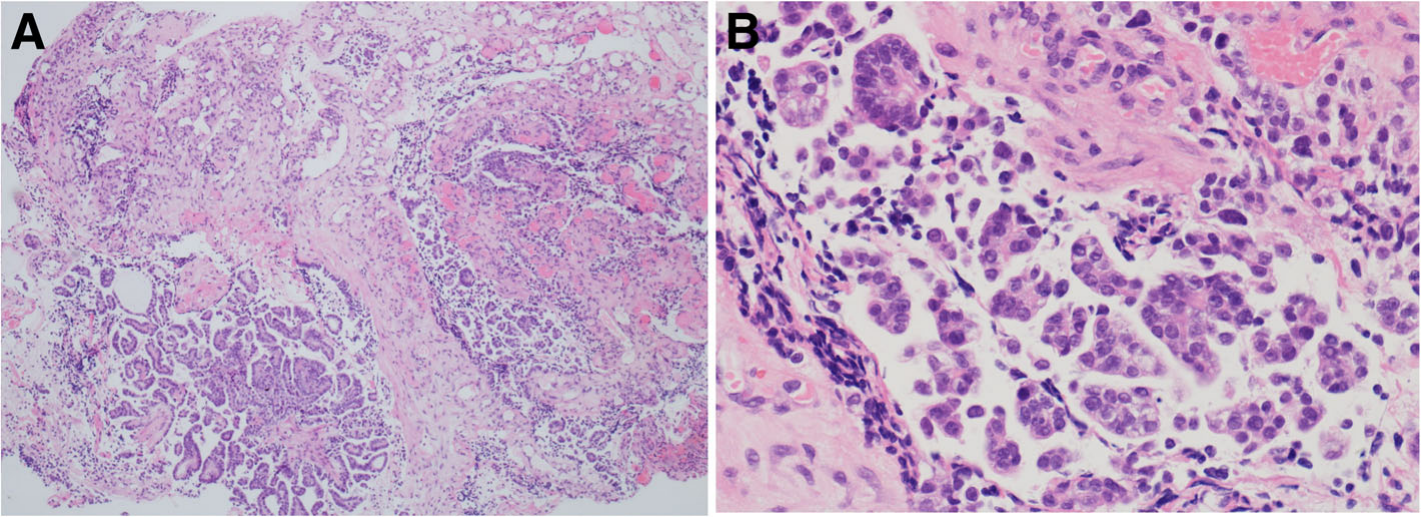
Excision of vaginal lesions on May 4, 2016 revealed atypical vaginal adenosis (hematoxylin and eosin staining, **a**, × 10; **b**, × 50)

**Fig. 3:**
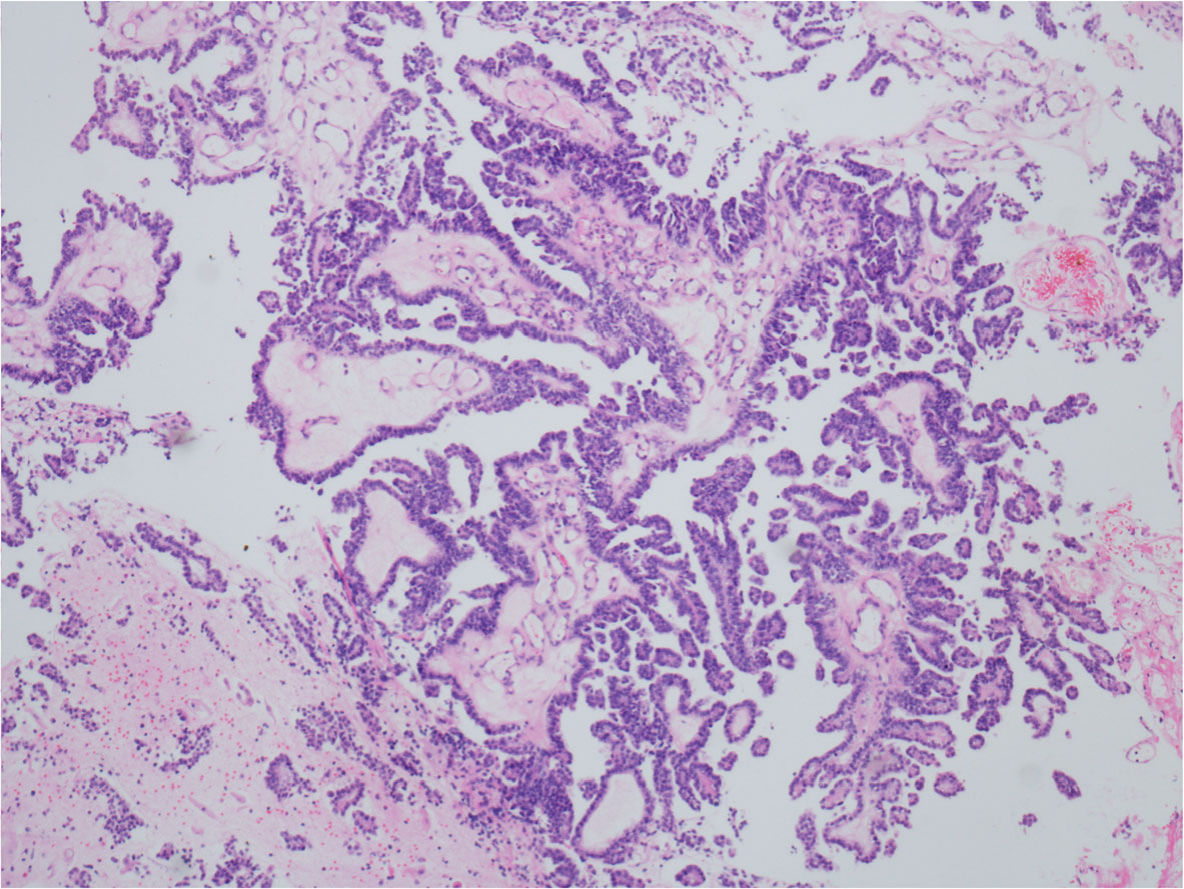
Biopsy of vaginal stump on April 15, 2017 revealed atypical vaginal adenosis (hematoxylin and eosin staining, × 10)

On December 24, 2015, the vaginal cytology results showed suspicious adenocarcinoma and atypical squamous epithelial cells. Another biopsy of the visible vaginal lesion suggested serous papillary glands with active growth. She underwent laparoscopic hysterectomy, bilateral salpingo-oophorectomy, and excision of the vaginal lesions on May 4, 2016. The postoperative pathology revealed atypical vaginal adenosis (Fig. 2). Twelve months after the hysterectomy, on May 15, 2017, her physical examination revealed polypoid tissue on the anterior vaginal wall, and vaginal biopsy revealed vaginal atypical adenosis (Fig. 3).

### Discovery and treatment of vaginal clear cell carcinoma (August 2017 to December 2017)

**Fig. 4:**
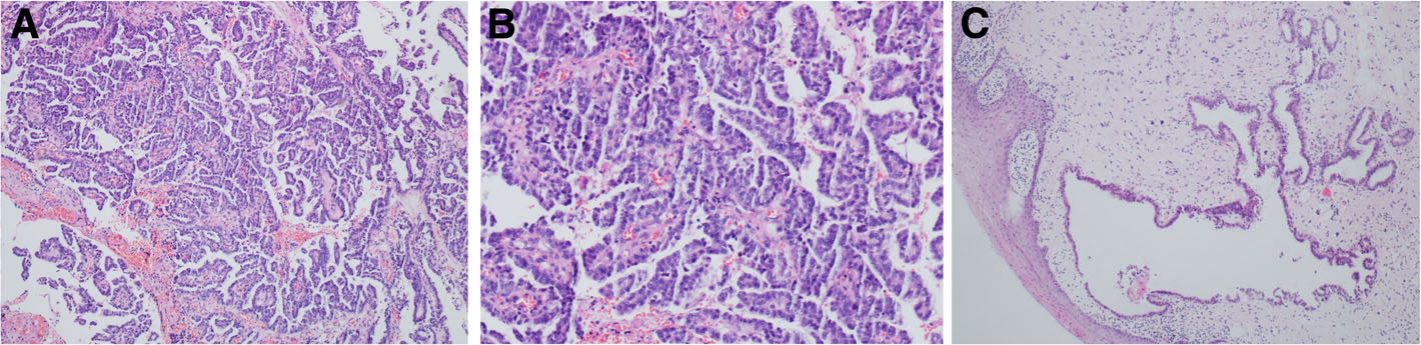
Excision of visible lesions in the mid-anterior vaginal wall on August 15, 2017 revealed clear cell carcinoma (hematoxylin and eosin staining, **a**, × 10; **b**, × 20) and coexisting atypical vaginal adenosis (hematoxylin and eosin staining, **c**, × 4)

On August 15, 2017, excision of the visible vaginal lesions revealed clear cell carcinoma of the vagina (Fig. 4a, b) and coexisting lesions of atypical adenomyosis (Fig. 4c). On September 15, 2017, she underwent wide local resection of the vagina, and the postoperative pathology results showed atypical vaginal adenosis with a negative margin and without residual carcinoma. Stage I vaginal clear cell carcinoma was confirmed. She underwent brachytherapy (30 Gy, five times) and concurrent cisplatin chemotherapy from October to December 2017. Since the patient refused external radiotherapy, concurrent cisplatin chemotherapy was applied only once (60 mg, intravenous). In October 2017, she provided samples for germline and somatic sequencing using a multi-gene panel of 57 gene mutations, including most genes involved in homologous recombination (HR) and non-HR pathways, such as *BRCA 1/2*, *RAD51C*, *PTEN*, *TP53*, *VHL*, *BAP1*, *SETD2*, *PBRM1*, and *MTOR*. No deleterious variants or variants of unknown significance were discovered.

### Follow-up (December 2017 to the present)

The patient participated in regular follow-up examinations. On July 18, 2018, she underwent a vulvar biopsy because of a vulvar ulcer. The pathological findings revealed inflammation, which improved after treatment with topical hormones. Her symptoms have since been relieved. Her progression-free survival of vaginal cancer reached 20 months in January 2019.

## Discussion

Primary vaginal malignancies are very rare, accounting for approximately 2% of all female genital malignancies [8]. More than 80% of vaginal cancers are squamous cell carcinomas [9]. Vaginal clear cell carcinoma is a rare type of vaginal cancer that usually occurs in women whose mothers used DES during pregnancy [10]. However, there have only been three known cases of vaginal clear cell carcinoma without prenatal DES exposure, most likely due to the malignant transformation of vaginal adenosis or atypical vaginal adenosis (Table 2) [5–7]. In the report by Uehara et al. [5], a 54-year-old woman complained of a 3-month history of genital bleeding, and the examination revealed clear cell adenocarcinoma at the anterior vagina, congenital anomalies of the bicornuate uterus and vaginal septum, and left ureteral agenesis. The patient was well without recurrence at 43 months after anterior pelvic exenteration. In the report by Satou et al. [6], another patient died of disease 16 months after radical hysterectomy and chemotherapy. In the report by Prasad et al. [7], the tumor, whose features were found to be similar to those of small cell carcinomas arising elsewhere in the female genital tract, was studied by light and electron microscopy and immunohistochemistry; intracytoplasmic electron-dense neurosecretory-type granules were observed, and immunohistochemistry revealed chromogranin A. The current report describes the fourth case, in which a definite evolution from vaginal adenosis to atypical vaginal adenosis and ultimately to clear cell carcinoma was observed.

**Table 2:** Cases of vaginal clear cell carcinoma due to the potential malignant transformation of vaginal adenosis or atypical vaginal adenosis without prenatal DES exposure in the English literature

Reference	Age at diagnosis of carcinoma	Atypical adenosis	Congenital anomalies	Courses from adenosis to carcinoma	Treatment	Disease-free survival	Overall survival	Recurrence	Mortality
Uehara T et al. [5]	54 years	None	Bicornuate uterus and vaginal septum and left ureteral agenesis	3 months	Anterior pelvic exenteration	43 months	43 months	No	No
Satou Y et al. [6]	38 years	None	Didelphys uterus, duplicated and imperforated vagina	Not available	Radical hysterectomy and chemotherapy	Not available	16 months	Not available	Yes
Prasad CJ [7]	34 years	Yes	None	Not available	Vaginectomy with bilateral inguinal lymph node dissection, chemotherapy of cisplatin and etoposide, teletherapy, brachytherapy	Not available	6 months	With tumor noted at deep margins of the vagina	Yes
Case in the report	45 years	Yes	None	6 years	Hysterectomy, wide local resection of vaginal lesion, and brachytherapy	16 months	16 months	No	No

The exact pathogenesis of the malignant transformation of vaginal adenosis without prenatal DES exposure is unknown. A study of clear cell carcinoma in women exposed prenatally to DES revealed the presence of both cervical ectropion and vaginal adenosis in all 20 specimens, and tubo-endometrial glands were intimately related to the carcinoma in 18 of the 20 cases, suggesting that the tubo-endometrial epithelium, whether in the ectocervix or vagina, serves as a source for the development of clear cell adenocarcinoma [11]. The frequency with which atypical tubo-endometrial glands in the vagina and cervix are associated with these carcinomas and the proximity of the former to the latter provide strong evidence that atypical vaginal adenosis and atypical cervical ectropion of the tubo-endometrial type are precursors of clear cell adenocarcinoma [12]. Lewis et al. [13] consistently found aneuploidy in 3 cases of invasive clear cell carcinoma of the vagina, suggesting that the immediate precursor state should also be in the aneuploid range. The 3 adenosis specimens, however, were in the normal diploid to tetraploid range. Aside from the toxicity of DES exposure, chemotherapeutic drugs may play a role in promoting the occurrence of vaginal adenosis and carcinoma. Cases of vaginal adenosis after topical 5-fluorouracil therapy for vaginal HPV-associated lesions [14] and vaginal adenosis together with clear cell carcinoma after 5-fluorouracil treatment for condylomas [15] have been reported. Congenital anomalies of the genitourinary tract have been suspected as the cause of clear cell carcinoma without DES exposure [5], which has been disputed [16]. Although there is a case report of adenocarcinoma originating from metanephric remnants [17], it is unlikely that it originated from clear cell carcinoma because of the topographical dissimilarity [18]. Currently, objective findings suggest that human prenatal epithelialization of the cervix and vagina results in 3 morphogenetically determined units [19], which may provide new insight into the histogenesis and transformation of vaginal adenosis.

In our report, before the discovery of adenosis, the patient had undergone multiple medical and invasive treatments, including treatment with tacrolimus and sirolimus, laser treatment and repeated biopsies. Whether these medical regimens and procedures would prompt the production of atypical adenosis or a transformation to vaginal cancer requires further exploration. Although there have been no reports on the relationship between trauma or medical treatments, except for diethylstilbestrol, and the transformation of adenosis, an off-label and unreasonable application of medicine should be avoided.

The natural history from vaginal adenosis to cancer varies greatly. Most patients with vaginal adenosis have no obvious symptoms. The lesions range widely, and symptoms can manifest as postcoital hemorrhage, sexual pain and a vaginal burning sensation [20]. In some cases, vaginal palpation reveals submucous nodular or sandy lesions 0.5–5 cm in diameter [20]. However, the main clinical manifestations of vaginal cancer include irregular vaginal bleeding, postpartum hemorrhage, postmenopausal hemorrhage and increased leucorrhea. The most common type of local vaginal lesions is the papillary or cauliflower type, followed by the ulcerative or infiltrative type. Difficulty in sexual intercourse is a typical symptom of advanced vaginal tumors.

Vaginal adenosis and clear cell carcinoma often occur several years after exposure to DES in the uterine cavity. Non-DES-induced vaginal adenosis has a reported incidence of approximately 10% in adult women. In the present case, the patient’s mother did not use DES during pregnancy since DES was never introduced into the Chinese market. Vaginal clear cell carcinoma was identified 6 years after the discovery of vaginal adenosis. A consensus regarding the detection and diagnosis of atypical vaginal adenosis and/or vaginal clear cell carcinoma is lacking. Cytology has been clinically valuable in proving cases of vaginal adenosis and adenocarcinoma [21]. Colposcopy with biopsy for abnormal vaginal and/or cervical cytology results could reveal possible lesions, as described in our report.

Laser therapy, cryotherapy and cautery can be used to treat superficial and small lesions of vaginal adenosis [22]. The lesions can also be coated topically with 10–20% silver nitrate or potassium dichromate solution for lesion necrosis and exfoliation. For a single localized submucosal lesion, complete resection of the lesion can be performed. For those with severe atypical hyperplasia or malignant transformation, the principle of treatment is the same as for those with vaginal cancer, despite a lack of sufficient evidence [23]. On the other hand, radiotherapy is the first choice for some patients with early or advanced vaginal cancer [24]. Radiotherapy includes brachytherapy and external beam. The use of brachytherapy in vaginal cancer imparts a benefit in terms of disease-specific and overall survival [25, 26]. The treatment of vaginal cancer with a multichannel cylinder produces high local control [27]. Surgery is also an option for patients with early-stage primary vaginal cancer [28]. Patients with early-stage vaginal tumors without deep infiltration may undergo radical hysterectomy, partial vaginal resection and pelvic lymphadenectomy. The margin of vaginal resection should be 2–3 cm beyond the tumor. For vaginal midsegment tumors, in addition to total vaginal hysterectomy, inguinal lymph node or pelvic lymph node resection should be performed according to the size of the lesion and the location of lymph node metastasis [29]. Total vaginal resection, including rectal resection or cystectomy (pelvic exenteration), is necessary for treatment, but the operation is extremely complicated [30, 31]. The effect of chemotherapy has been shown to be minimal. In the case reported by Satou et al. [6], the patient survived only 16 months after radical hysterectomy and chemotherapy. However, in the current case, several inappropriate therapy protocols were applied. Before being transferred to our unit, the patient was treated with tacrolimus and sirolimus, neither of which had definite indications or resulted in symptomatic relief. Although there have been several reports on the application of tacrolimus for the treatment of erosive lichen planus [32–34], these experiences are not applicable to the treatment of adenosis.

In conclusion, we report the fourth case in the world of vaginal clear cell carcinoma stemming from the malignant transformation of vaginal adenosis without prenatal DES exposure, with serial evidence of oncological evolution. Wide local excision with radiotherapy provided at least 16 months of disease-free survival. Serial follow-up examinations with vaginal cytology is essential for patients with vaginal adenosis for the diagnosis of atypical lesions and even cancer.

## Data Availability

The medical history of this patient, including detailed procedures for diagnosis and treatment, are listed in Table 1 and described in the “Case Presentation” section.

## Abbreviations

ASC-H: Atypical squamous cells, cannot exclude high-grade squamous intraepithelial lesions
DES: Prenatal diethylstilbestrol
HPV: Human papillomavirus
HR: Homologous recombination
HSIL: High-grade squamous intraepithelial lesion
